# Orai3 Surface Accumulation and Calcium Entry Evoked by Vascular Endothelial Growth Factor

**DOI:** 10.1161/ATVBAHA.115.305969

**Published:** 2015-08-26

**Authors:** Jing Li, Alexander-Francisco Bruns, Bing Hou, Baptiste Rode, Peter J. Webster, Marc A. Bailey, Hollie L. Appleby, Nicholas K. Moss, Judith E. Ritchie, Nadira Y. Yuldasheva, Sarka Tumova, Matthew Quinney, Lynn McKeown, Hilary Taylor, K. Raj Prasad, Dermot Burke, David O’Regan, Karen E. Porter, Richard Foster, Mark T. Kearney, David J. Beech

**Affiliations:** From the Leeds Institute of Cardiovascular and Metabolic Medicine, School of Medicine (J.L., A.-F.B., B.H., B.R., P.J.W., M.A.B., H.L.A., N.K.M., J.E.R., N.Y.Y., S.T., M.Q., L.M., H.T., K.E.P., D.J.B.) and School of Chemistry (R.F.), University of Leeds, Leeds, United Kingdom; Departments of Hepatobiliary and Transplant Surgery (K.R.P.) and Colorectal Surgery (D.B.), St. James’s University Hospital, Leeds, United Kingdom; and Yorkshire Heart Centre, Leeds General Infirmary, Leeds, United Kingdom (D.O.R.).

**Keywords:** calcium, cytosol, endothelial cells, Orai3 protein, vascular endothelial growth factor A

## Abstract

Vascular endothelial growth factor (VEGF) acts, in part, by triggering calcium ion (Ca^2+^) entry. Here, we sought understanding of a Synta66-resistant Ca^2+^ entry pathway activated by VEGF.

**Approach and Results—:**

Measurement of intracellular Ca^2+^ in human umbilical vein endothelial cells detected a Synta66-resistant component of VEGF-activated Ca^2+^ entry that occurred within 2 minutes after VEGF exposure. Knockdown of the channel-forming protein Orai3 suppressed this Ca^2+^ entry. Similar effects occurred in 3 further types of human endothelial cell. Orai3 knockdown was inhibitory for VEGF-dependent endothelial tube formation in Matrigel in vitro and in vivo in the mouse. Unexpectedly, immunofluorescence and biotinylation experiments showed that Orai3 was not at the surface membrane unless VEGF was applied, after which it accumulated in the membrane within 2 minutes. The signaling pathway coupling VEGF to the effect on Orai3 involved activation of phospholipase Cγ1, Ca^2+^ release, cytosolic group IV phospholipase A2α, arachidonic acid production, and, in part, microsomal glutathione S-transferase 2, an enzyme which catalyses the formation of leukotriene C_4_ from arachidonic acid. Shear stress reduced microsomal glutathione S-transferase 2 expression while inducing expression of leukotriene C_4_ synthase, suggesting reciprocal regulation of leukotriene C_4_–synthesizing enzymes and greater role of microsomal glutathione S-transferase 2 in low shear stress.

**Conclusions—:**

VEGF signaling via arachidonic acid and arachidonic acid metabolism causes Orai3 to accumulate at the cell surface to mediate Ca^2+^ entry and downstream endothelial cell remodeling.

## Abstract 2

Supplemental Digital Content is available in the text.

The 3 mammalian Orais are tetraspanin-like proteins of Ca^2+^-permeable plasma membrane channels.^1^ The most studied is Orai1, a key player in the Ca^2+^ release–activated Ca^2+^ (CRAC) channels of T cells.^1^ An activator of Orai1 is the stromal interaction molecule 1 (STIM1), which confers a link between Ca^2+^ from the endoplasmic reticulum and Orai1-dependent channels at the plasma membrane.^2^ The related Orai3 is also capable of generating CRAC channel-like currents when overexpressed in cell lines, as is native Orai3 in estrogen receptor positive breast cancer cell lines.^3,4^ However, endogenous Orai3 does not contribute to store depletion-evoked Ca^2+^ entry in vascular cell types.^5,6^ Intriguing studies suggest that Orai3 importantly contributes to separate but related Ca^2+^ entry channels operating independently of store depletion: arachidonic acid (AA)–regulated Ca^2+^ channels.^7^ Such channels exist in cell lines, primary acinar cells, vascular smooth muscle cells and taste bud cells^7–9^ and arise from heteromers of Orai1 and Orai3.^10,11^ Studies in HEK 293 cells suggest plasma membrane STIM1 is essential for AA–regulated Ca^2+^ channel activation.^12–14^ In HEK 293 cells, AA acts as an activator depending on the N-terminus of Orai3.^15^ Studies of vascular smooth muscle cells suggest the contribution of Orai3 to leukotriene C_4_ (LTC4) regulated Ca^2+^ channels.^16–18^ These channels rely on an interaction with endoplasmic reticulum–resident STIM1^17,18^ and are activated by the AA metabolite LTC4 generated through the 5-lipoxygenase pathway by LTC4 synthase (LTC4S).^19^

Vascular endothelial growth factor (VEGF) is a primary inducer of endothelial cell function, for example in the regulation of vascular permeability and angiogenesis.^20^ Intracellular Ca^2+^ elevation is an early event in the action of VEGF acting through VEGF receptor-2 (VEGFR2).^21,22^ It arises because of Ca^2+^ release from intracellular stores and multiple types of Ca^2+^ entry that sustain the cytosolic Ca^2+^ elevation.^23,24^ One type of Ca^2+^ entry occurs through endogenous CRAC-like channels because it is partially suppressed by knockdown of Orai1 or STIM1 and by a small molecule inhibitor of CRAC channels, Synta66 (S66).^22^ S66 has been studied for effects on >50 ion channels, receptors, transporters, and calcium release mechanisms and no significant effects were observed; it seems to be a specific inhibitor of CRAC channels.^6,22,25,26^ However, there is also an Orai1/STIM1-dependent signal that is resistant to CRAC channel blockade.^22^ Therefore, Orai1 and STIM1 contribute not only to a CRAC channel but also to a pharmacologically distinct S66-resistant Ca^2+^ entry channel. Here, we investigated the role of Orai3 and the activation mechanism for these S66-resistant channels.

## Materials and Methods

Materials and Methods are available in the online-only Data Supplement.

## Results

### Orai3 Mediates VEGF-Evoked Ca^2+^ Entry in HUVECs

**Figure 1.**
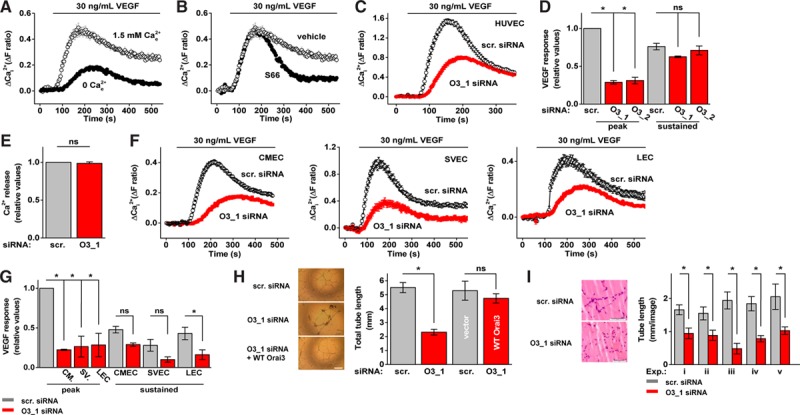
Role of Orai3 in vascular endothelial growth factor (VEGF)–evoked Ca^2+^ entry and endothelial cell remodeling. **A**, Example responses to VEGF stimulation (30 ng/mL) from human umbilical vein endothelial cells (HUVECs) in the presence or absence of Ca^2+^ (n=4/N=16). **B**, Example VEGF responses from HUVECs in the presence of Synta66 (S66; (5 μmol/L) or vehicle control (n=3/N=32 each). **C**, Example responses to VEGF stimulation (30 ng/mL) from HUVECs treated with scrambled (scr.) or Orai3_1 (O3_1) siRNA (n=3/N=60) each. **D**, Mean VEGF responses from HUVECs at peak or sustained (330 s) for cells transfected with scrambled (scr.), Orai3_1 (O3_1) or Orai3_2 (O3_2) siRNA (n=3/N=32 each). **E**, As for (**C**) but in the absence of extracellular Ca^2+^ (n=3/N=64 each). **F**, Example responses to VEGF stimulation (30 ng/mL) from cardiac microvascular endothelial cells (CMECs ; n=3/N=48 each), saphenous vein endothelial cells (SVECs; n=3/N=24 each), and liver endothelial cells (LECs; n=5/N=25 each). **G**, Mean data and analysis of data from CMECs (CM.), SVECs (SV.), and LECs as exemplified in (**F**). **H**, In vitro tube lengths of HUVECs after transfection with scr. or O3_1 siRNA; or scr. compared with O3_1 plus wild-type (WT) Orai3 clone (n=3 each). Scale bar, 50 μm. **I**, In vivo tube length of HUVECs in mice after transfection with scr. or O3_1 siRNA (5 independent experiments: i–v). Scale bars, 50 μm. Data are represented as mean±SEM. *P<0.05; not significant (ns) *P*>0.05.

In the absence of extracellular Ca^2+^, VEGF (30 ng/mL) caused a Ca^2+^ release event, which reached a maximum in 3 minutes and then decayed (Figure 1A). In the presence of physiological Ca^2+^, VEGF caused greater elevation of intracellular Ca^2+^, which reached a maximum in 2 minutes (Figure 1A). CRAC channel inhibition by S66 had no effect on the rising phase of this Ca^2+^ elevation but suppressed the later sustained phase (Figure 1B). To investigate the role of Orai3, we performed siRNA-mediated knockdown using 2 different siRNAs that reduced the abundance of Orai3 without affecting expression of Orai1, Orai2, STIM1, or STIM2 or the STIM1-regulated TRPC1, TRPC4, or TRPC5 channels (Figure IA–IE in the online-only Data Supplement). Knockdown of Orai3 reduced the VEGF-evoked Ca^2+^ entry by 69% to 72% at its peak and 7% to 18% at the sustained phase measured at 330 s (Figure 1C and 1D). Ca^2+^ release was unaffected (Figure 1E). The data suggest importance of Orai3 in the early phase of VEGF-evoked Ca^2+^ entry.

### Orai3 Mediates VEGF-Evoked Ca^2+^ Entry in a Variety of Human Endothelial Cell Types

To address the relevance to other endothelial cells, we first studied human cardiac microvascular endothelial cells. There was robust Orai3-dependent Ca^2+^ elevation in response to VEGF, similar to that in human umbilical vein endothelial cells (HUVECs; Figure 1F and 1G). To determine the relevance to patients, we isolated endothelial cells from saphenous vein obtained at coronary artery bypass grafting. Again a similar Orai3-dependent VEGF response was observed (Figure 1F and 1G). We also isolated sinusoidal endothelial cells from normal liver tissue obtained at resection for colorectal liver metastases: the VEGF response was similar to that of HUVECs and had similar Orai3-dependence (Figure 1F and 1G). The data suggest that Orai3 is a significant contributor to VEGF signaling in several important vascular settings: macrovascular as well as microvascular, and in patients with coronary artery disease and cancer.

### Orai3 Positively Affects VEGF-Induced Endothelial Cell Remodeling

To investigate functional consequences of Orai3 in this context, we first performed transwell migration and cell count assays with HUVECs treated with Orai3 siRNA. Orai3 depletion suppressed both migration and proliferation (Figure IIA and IIB in the online-only Data Supplement). We, therefore, investigated tube formation in coculture with fibroblasts and on Matrigel in vitro and in vivo in mice. Orai3 siRNA reduced tube length and number of tube branches (Figure 1H and 1I; SIIC and IID). Wild-type Orai3 cDNA rescued tube formation, consistent with Orai3 siRNA generating its effect through Orai3 suppression rather than an off-target mechanism (Figure 1H). The data suggest Orai3 as a positive factor in VEGF-induced endothelial cell remodeling.

### Exogenous AA Causes Orai3-Dependent Ca^2+^ Entry

**Figure 2.**
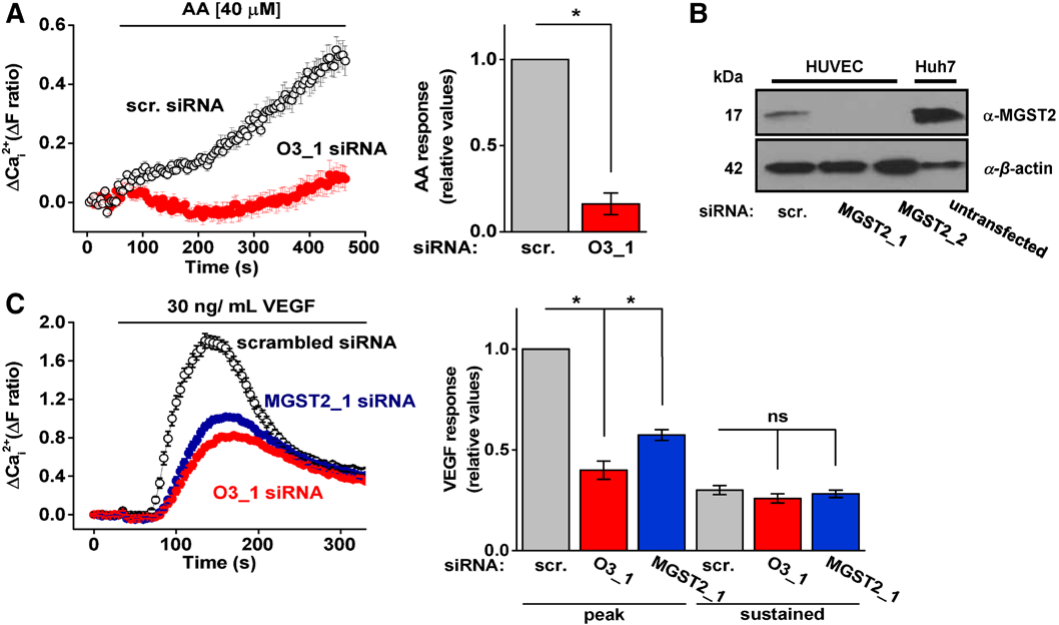
Exogenous arachidonic acid (AA) evokes Orai3-dependent Ca^2+^ entry and microsomal glutathione S-transferase 2 (MGST2) is required in the action of vascular endothelial growth factor (VEGF). **A**, Example responses and mean data of human umbilical vein endothelial cells (HUVECs) exposed to exogenous AA (40 μmol/L). All cells were pretreated with thapsigargin (2 μmol/L) and studied in the presence of S66 (5 μmol/L). Cells were transfected with scrambled (scr.) or Orai3 (O3_1) siRNA; (n=3/N=60 each). **B**, Representative immunoblot of MGST2 in HUVECs transfected with scr. or MGST2 siRNA_1 or MGST2 siRNA_2. Untransfected Huh7 cells were used as a positive control for MGST2 expression. **C**, Example VEGF responses and mean data from HUVECs transfected with scr., O3_1 or MGST2_1 siRNA. Mean data are for VEGF responses at peak or after 330 s (n=3/N=36 each). Data are represented as mean±SEM; *P<0.05; ns *P*>0.05.

A downstream mechanism of VEGFR2 phosphorylation is the activation of PLCγ1 (phospholipase C γ1) leading to the production of AA by cytosolic group IV phospholipase A2α (cPLA2α; Figure IIIA in the online-only Data Supplement),^27–29^ which has been previously linked to AA–regulated Ca^2+^ channels.^30^ The PLC inhibitor U73122 abolished VEGF-evoked Ca^2+^ elevation.^21,22^ Edelfosine, an alternative PLC inhibitor, had the same effect (Figure IIIB in the online-only Data Supplement). Inhibitors of several other signaling elements downstream of VEGFR2 were tested and had no effect (Figure IIIB in the online-only Data Supplement). Because AA production is downstream of PLCγ1, we investigated if it was possible to circumvent VEGFR2 by directly applying exogenous AA. The response to exogenous AA included Ca^2+^ release (Figure IIIC and IIID) and so we investigated if there was an effect on Ca^2+^ entry independent of release and CRAC channel activation by first depleting Ca^2+^ stores with thapsigargin and including S66. Ca^2+^ was present in the extracellular medium. There was robust Ca^2+^ entry in response to 40 μmol/L AA. Lower AA concentrations generated only small inconsistent effects (Figure IIIE in the online-only Data Supplement). To investigate if 40 μmol/L AA caused nonspecific membrane disruption, we applied 40 μmol/L eicosatetraynoic acid, a nonmetabolizeable AA analog, which did not cause Ca^2+^ entry (Figure IIIF in the online-only Data Supplement). Moreover, Orai3 siRNA strongly suppressed the AA-evoked Ca^2+^ entry, suggesting that exogenous AA activates the Orai3 mechanism without causing membrane disruption (Figure 2A). The data are consistent with AA being a component of the pathway between VEGF and Orai3 but suggest that AA has relatively weak potency in the absence of cofactors triggered by VEGF.

### Role of MGST2

We investigated whether AA metabolites are involved in the action of VEGF. It was previously suggested that HUVECs generate AA metabolites, such as LTC4 by enzymatic activity of microsomal glutathione S-transferase 2 (MGST2).^31,32^ We confirmed the expression of MGST2 and knocked down its expression by 2 siRNAs (Figure 2B; Figure IIIG in the online-only Data Supplement). Importantly, there was significant reduction in VEGF-evoked Ca^2+^ entry after MGST2 depletion and the character of the effect of MGST2 depletion was similar to that of Orai3 depletion (Figure 2C). Metabolism of AA by the cyclooxygenase pathway was not involved because indomethacin (10 μmol/L)^33^ had no effect on VEGF-evoked Ca^2+^ entry (Figure IIIH in the online-only Data Supplement). The data suggest a role for AA metabolism by MGST2 in VEGF-evoked Orai3-dependent Ca^2+^ entry.

### VEGF Is Required for Orai3 Plasma Membrane Localization

**Figure 3.**
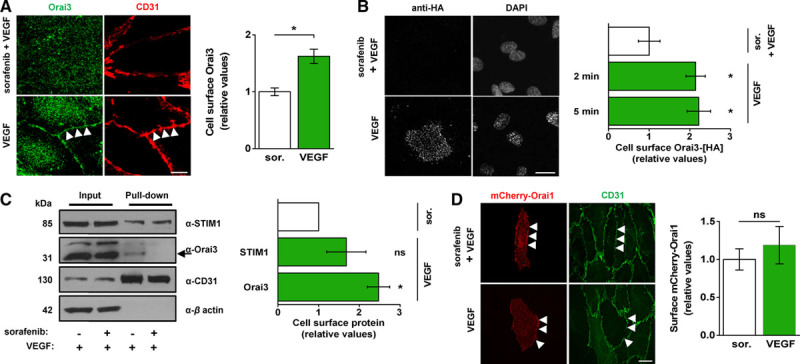
Selective Orai3 plasma membrane accumulation is evoked by vascular endothelial growth factor (VEGF). **A**, Representative images and mean data of cells treated with sorafenib (1 μmol/L) or vehicle (DMSO [dimethyl sulfoxide]) before stimulation with VEGF (30 ng/mL) for 5 minutes. Cells were labeled with anti-Orai3 antibody (Orai3, green) and anti-CD31 antibody (CD31, red). Scale bar, 2 μm. Arrows point to example cell perimeter as indicated by CD31 labeling. Mean data shows cell-surface Orai3 (n=6/N=18 each). **B**, Representative images and mean data of human umbilical vein endothelial cells (HUVECs) overexpressing Orai3-[HA] and treated as in (**A**). Cells were labeled with the anti-HA antibody. Scale bar, 10 μm. VEGF was applied for 5 minutes (n=3/N=15 each) or 2 minutes (n=3/N=45 each). **C**, Representative immunoblot and mean data from 3 experiments for cells treated as in (**A**) before biotinylation. The arrow points to Orai3 labeled by anti-Orai3 antibody (α-Orai3). The protein band above it, labeled nonspecifically by α-Orai3, has unknown identity. Where indicated (+), sorafenib (sor.) and VEGF were used at 1 μmol/L and 30 ng/mL, respectively. VEGF was applied for 5 minutes. **D**, Representative images and mean data for mCherry-Orai1 surface localization in cells treated as in (**A**); (sor., n=7/N=29; VEGF, n=4/N=14). Scale bar, 10 μm. All data are from HUVECs. Data are represented as mean±SEM; *P<0.05; ns *P*>0.05.

For Orai3 to contribute to a Ca^2+^ entry channel, it has to localize to the plasma membrane. However, we could not detect it at the plasma membrane under basal conditions. We, therefore, tested if Orai3 was at the plasma membrane after stimulation with VEGF. Cells in the control group were treated with sorafenib (1 μmol/L), a multikinase inhibitor, to suppress constitutive ligand-independent VEGF receptor signaling. Co-staining for CD31 protein defined the location of plasma membrane Orai3. Importantly, we could only detect Orai3 at the plasma membrane after VEGF stimulation (Figure 3A). To further explore the phenomenon, we generated a functional Orai3 construct with a hemagglutinin (HA) epitope tag in the second extracellular loop (Orai3-[HA]; Figure IVA–IVC in the online-only Data Supplement), which allowed detection of overexpressed surface-localized Orai3 in nonpermeabilized cells. Again, Orai3 was only at the plasma membrane after VEGF stimulation (Figure 3B). Surface accumulation of Orai3 in response to VEGF was rapid, occurring within 2 minutes after VEGF application (Figure 3B), which aligns well with the time course of the Ca^2+^ elevation (Figure 1A and 1B). Surface biotinylation experiments confirmed these results for endogenous Orai3 (Figure 3C). STIM1 was by contrast constitutively at the plasma membrane and not significantly increased by VEGF (Figure 3C). Orai1 was likewise at the membrane and not affected by VEGF (Figure 3D). The data suggest that VEGF triggers Orai3 accumulation at the plasma membrane to enable Orai3-dependent Ca^2+^ entry.

### Roles of AA and MGST2 in Membrane Accumulation of Orai3

**Figure 4.**
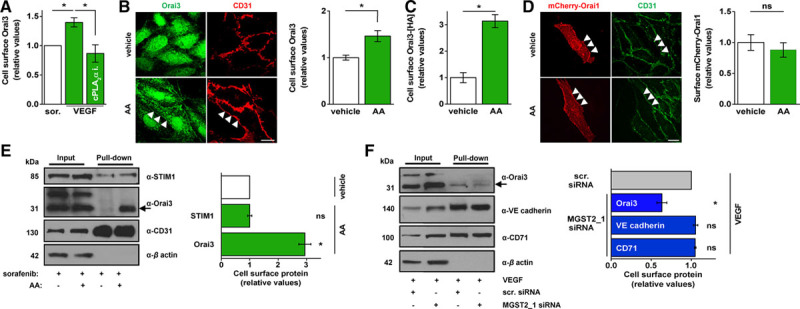
Selective Orai3 plasma membrane accumulation is evoked by exogenous arachidonic acid (AA) and depends on microsomal glutathione S-transferase 2 (MGST2). **A**, Mean immunofluorescence data for α-Orai3-labeled Orai3 in cells treated with sorafenib (1 μmol/L), vehicle or cytosolic group IV phospholipase A2α (cPLA2α) inhibitor (1 μmol/L) before stimulation with vascular endothelial growth factor (VEGF; 30 ng/mL) for 10 minutes (n=4/N=15 each). **B**, Representative images and mean data (n=4/N=22 each) of cells treated with sorafenib before stimulation with vehicle or AA (40 μmol/L) for 10 minutes. Cells were labeled with anti-Orai3 (green) and anti-CD31 (red) antibodies. Scale bar, 10 μm. Arrows point to cell perimeter as indicated by signal for CD31. **C**, Mean data for cells overexpressing Orai3-[HA] and treated as in (**B**; n=3/N=15 each). **D**, Representative images and mean data for mCherry-Orai1 surface localization in cells treated as in (**B**; vehicle control, n=7/N=29; AA, n=3/N=16 each). Scale bar, 10 μm. **E**, Representative immunoblot and mean data from 3 experiments for cell surface Orai3 from cells treated as in (**B**) before biotinylation. The arrow points to Orai3 labeled by anti-Orai3 antibody (α-Orai3). The protein band above it, labeled nonspecifically by α-Orai3, has unknown identity. Where indicated (+), sorafenib and AA were used at 1 μmol/L and 40 μmol/L, respectively. AA was applied for 5 minutes. **F**, Representative immunoblot and mean data from 3 experiments for cells treated with VEGF (30 ng/mL) for 5 minutes before biotinylation. The arrow points to Orai3 labeled by anti-Orai3 antibody (α-Orai3). The protein band above it, labeled nonspecifically by α-Orai3, has unknown identity. Where indicated (+), cells were transfected with control scrambled (scr.) siRNA or MGST2 siRNA_1. All data are from HUVECs. Data are represented as mean±SEM. STIM1 indicates stromal interaction molecule 1. *P<0.05; not significant (ns) *P*>0.05.

To elucidate a mechanism for the surface accumulation of Orai3, we investigated cPLA_2_α, which generates AA in response to VEGF.^27–29^ Importantly, inhibition of cPLA_2_α prevented VEGF-induced plasma membrane accumulation of Orai3 (Figure 4A). Furthermore, exogenous AA caused Orai3 surface accumulation (Figure 4B and 4C). Orai1 was not affected by AA (Figure 4D). Similarly, STIM1 was constitutively present at the plasma membrane and not affected by AA (Figure 4E). In addition, knockdown of MGST2 reduced VEGF-dependent surface localization of Orai3, whereas surface marker proteins, VE cadherin and CD71, were unaffected (Figure 4F). The data suggest that VEGF evokes surface accumulation of Orai3 via cPLA_2_α, AA and, in part, the metabolism of AA by MGST2.

### Reciprocal Regulation of *MGST2* and *LTC4S* Gene Expression by Shear Stress

**Figure 5.**
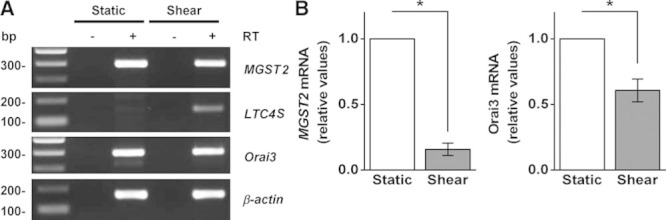
Analysis of *MGST2*, *LTC4S*, and *Orai3* gene expression in human umbilical vein endothelial cell (HUVEC). **A**, Example agarose gel for end-point polymerase chain reaction products obtained with primers for *MGST2*, *LTC4S*, *Orai3*, and β-actin from HUVEC cDNA. Cells were exposed to static and shear-stress conditions, respectively, before harvesting mRNA and reverse transcriptase reaction (+RT [reverse transcriptase]) to generate cDNA. −RT denotes control reaction. **B**, Mean data and analysis of *MGST2* and *Orai3* mRNA (n=6). Data are represented as mean±SEM; *P<0.05.

Although endothelial cells exist without shear stress during early stages of embryonic and adult angiogenesis and in low or disturbed shear stress in mature vessels, shear stress is a force constantly experienced by many endothelial cells and a driver for vascular maturation, endothelial cell alignment, and other vascular phenomena.^34^ We, therefore, investigated the effect of shear stress on expression of the *MGST2* gene. Expression of *MGST2* was reduced while not abolished by shear stress (Figure 5A and 5B). The effect on *Orai3* gene expression was similar (Figure 5A and 5B). Consistent with previous work^32^ in static conditions, we could not detect expression of *LTC4S* gene, an alternative mechanism for generating LTC4, but shear stress induced expression of *LTC4S* (Figure 5A). The data suggest a greater role for MGST2 in low shear stress conditions and a reciprocal effect of shear stress on the expression of *MGST2* and *LTC4S* genes.

## Discussion

This study shows relevance of Orai3 to VEGF signaling and downstream endothelial cell remodeling. It also shows a previously unrecognized mechanism for acute control over Ca^2+^ entry by Orai proteins. The data suggest that Orai3 is not constitutively at the plasma membrane but that it rapidly accumulates in the membrane in response to VEGF. Induced accumulation effectively serves as an activation mechanism. Orai1 and STIM1 are not similarly regulated: we find that they are constitutively localized to the plasma membrane, which is consistent with previous reports.^14,18,35^ We suggest that VEGF-evoked accumulation of Orai3 depends on PLCγ1 activation, subsequent Ca^2+^ release that activates cPLA_2_α, catalysis of the production of AA and then metabolism of this AA, in part, by MGST2 to generate metabolites, such as LTC4. We hypothesize that a combination of AA itself and AA metabolites such as LTC4 act on Orai3 to cause its surface accumulation and its activation (if it is not already constitutively active).

It is surprising that Orai3 lacks localization to the plasma membrane in endothelial cells under basal conditions. First, it contrasts with the situation for Orai1, as shown in this study and observed previously.^22^ Second, overexpression of Orai3 in the HEK 293 cell line, a commonly used mammalian cell expression system, leads to constitutive Orai3 at the plasma membrane as shown by previous studies^36^ and confirmed by us (Figure IVD in the online-only Data Supplement). By contrast, we found no evidence for similar localization of endogenous Orai3 in endothelial cells. There is clearly a technical challenge in studying endogenous low abundance membrane proteins such as Orai3 and so, while we confirmed the specificity of our anti-Orai3 antibody for studies of endogenous Orai3 in endothelial cells (Figure ID in the online-only Data Supplement), it was important to test our hypothesis without using this antibody. For this work, we expressed exogenous HA-tagged Orai3 in endothelial cells but we were careful to use the minimum expression abundance required for detection, making observations only 6 hours after transfection to reduce the likelihood of overexpression and thus artificial bias of Orai3 to the plasma membrane.

The reason why there is basal exclusion of Orai3 from the surface membrane is unknown but we speculate that it is important to avoid incorporation of constitutively active Orai3-containing channels that could cause long term, potentially damaging, leak of Ca^2+^ into the cells. Whatever the reason, it presents a mechanistic challenge because, unlike Orai3, STIM1, and Orai1 are constitutively at the plasma membrane, yet, like Orai3, they both contribute to S66-resistant Ca^2+^ entry.^22^ This raises a question about how Orai3 integrates with or otherwise influences Orai1 and STIM1. We hope to reveal understanding of the mechanisms through future studies.

Studies of other cell types have shown dependence of Orai3 mechanisms not only on Orai1 and STIM1 but also AA and AA metabolism.^7,18,37^ It is notable that all studies have used a relatively high concentration of exogenous AA in efforts to mimic the effect of an endogenous receptor agonist. To generate a robust response we needed to use 40 μmol/L AA. Other studies have used ≤10× less, but such concentrations are still relatively high. This requirement could suggest that exogenous AA is inefficient at mimicking AA generated enzymatically inside cells or that a cofactor is required to activate the mechanism efficiently; activation of VEGFR2 by VEGF generates many signaling factors, some of which might synergize with AA.

We suggest a Ca^2+^ release-dependent mechanism for activation of this Orai3 system: Ca^2+^ release activating cPLA_2_α to catalyze the production of AA. This should not be taken to mean that Orai3 is activated by store depletion in these cells, because it is not (Figure V in the online-only Data Supplement). Physiological Ca^2+^ release does not necessarily cause Ca^2+^ store depletion. Our previous work on growth factor–activated Ca^2+^ signaling in vascular smooth muscle cells^35^ and measurements of stored Ca^2+^ in endothelial cells during agonist exposure^38^ have suggested that stores are efficiently maintained replete in the face of Ca^2+^ release because of reuptake of Ca^2+^ into stores via smooth endoplasmic reticulum Ca^2+^ ATPase. Store depletion is a stress to endoplasmic reticulum and so cells will have evolved mechanisms to minimize such stress during physiological signaling.

The reciprocal relationship between expression of this system and shear stress suggests that it may have greatest functional importance at sites with low or disturbed shear stress. Such sites occur in embryonic development but also at vascular loci in the adult that are vulnerable to disease or directly involved in on-going disease. Our detection of this Orai3 mechanism in endothelial cells from patients with coronary artery disease and cancer suggests relevance in pathophysiological settings; in support of this, atherosclerosis, tissue injury, cancer, and other related conditions have all been associated with increased PLA_2_ activity and AA.^39–42^

## Sources of Funding

This study was supported by research grants from the Medical Research Council, the Wellcome Trust, and British Heart Foundation. B. Hou was supported by a scholarship from the University of Leeds and the China Scholarship Council. P.J. Webster and J.E. Ritchie had fellowships from the LTHT (Leeds Teaching Hospitals Trust) Charitable Foundation and Cancer Research UK, respectively.

## Disclosures

None.

## Supplementary Material

## Significance

Although Ca^2+^ entry is known to mediate cellular responses to vascular endothelial growth factor (VEGF), there are multiple mechanisms and not all of them are defined or understood. Here, we describe a pathway for VEGF-evoked Ca^2+^ entry that surprisingly requires Orai3-containing channels. Moreover, it requires rapid accumulation of these channels at the plasma membrane in response to VEGF. Without VEGF, Orai3 is not at the membrane. Intriguingly, the signaling pathway required for Orai3 accumulation involves the enzyme microsomal glutathione S-transferase 2, a catalyst for arachidonic acid metabolism not previously associated with VEGF signaling. The Orai3 drives endothelial cell remodeling and so these mechanisms may be important in cardiovascular and cancer disease processes.
