# Supplementary material for: Orai3 Surface Accumulation and Calcium Entry Evoked by Vascular Endothelial Growth Factor
Source: Arterioscler Thromb Vasc Biol. 2015 Aug 26;35(9):1987–94. doi: 10.1161/ATVBAHA.115.305969 (PMC4548547; doi:10.1161/ATVBAHA.115.305969)
Supplement: Supplementary file 1 [file atv-35-1987-s001.pdf]

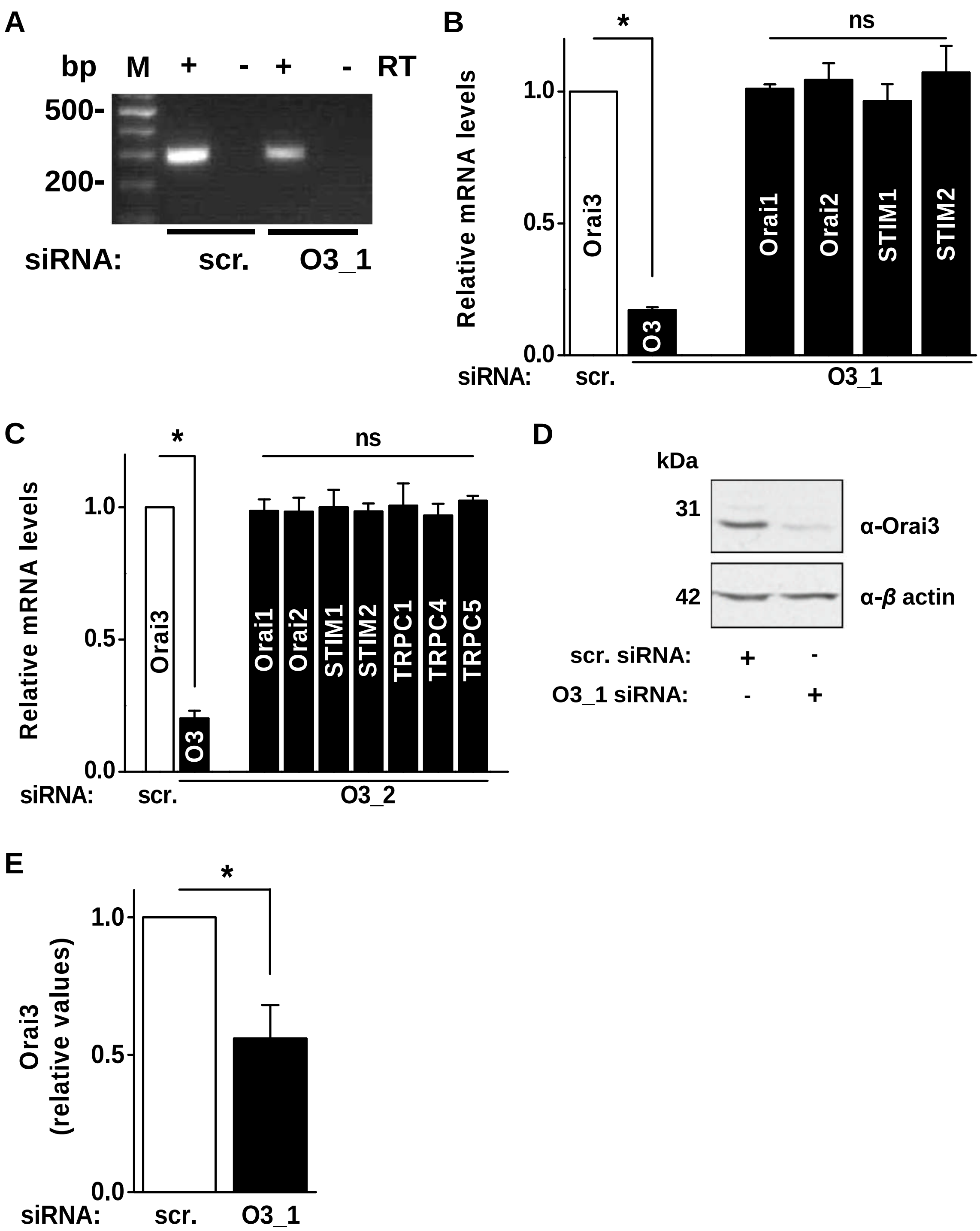

Supplemental Figure I. Validation of Orai3 expression and its specific knock down.

(A) Knock-down of Orai3 mRNA in HUVECs by Orai3 siRNA_1 compared with the scrambled (scr.) siRNA control.

(B) Quantitative real-time RT-PCR analysis of Orai3, Orai1, Orai2, STIM1 and STIM2 mRNAs from HUVEC transfected with scr. siRNA or Orai3 siRNA (O3_1); (n=3 for each).

(C) As for **(B)** but using Orai3 siRNA_2 (O3_2).

(D) Representative immunoblot for Orai3 from HUVEC transfected with scr. or Orai3 siRNA (O3_1).

(E) Mean data for experiments of the type exemplified in **(D)** (n=3 each).

Data are represented as mean ± SEM; *p < 0.05, ns p > 0.05.

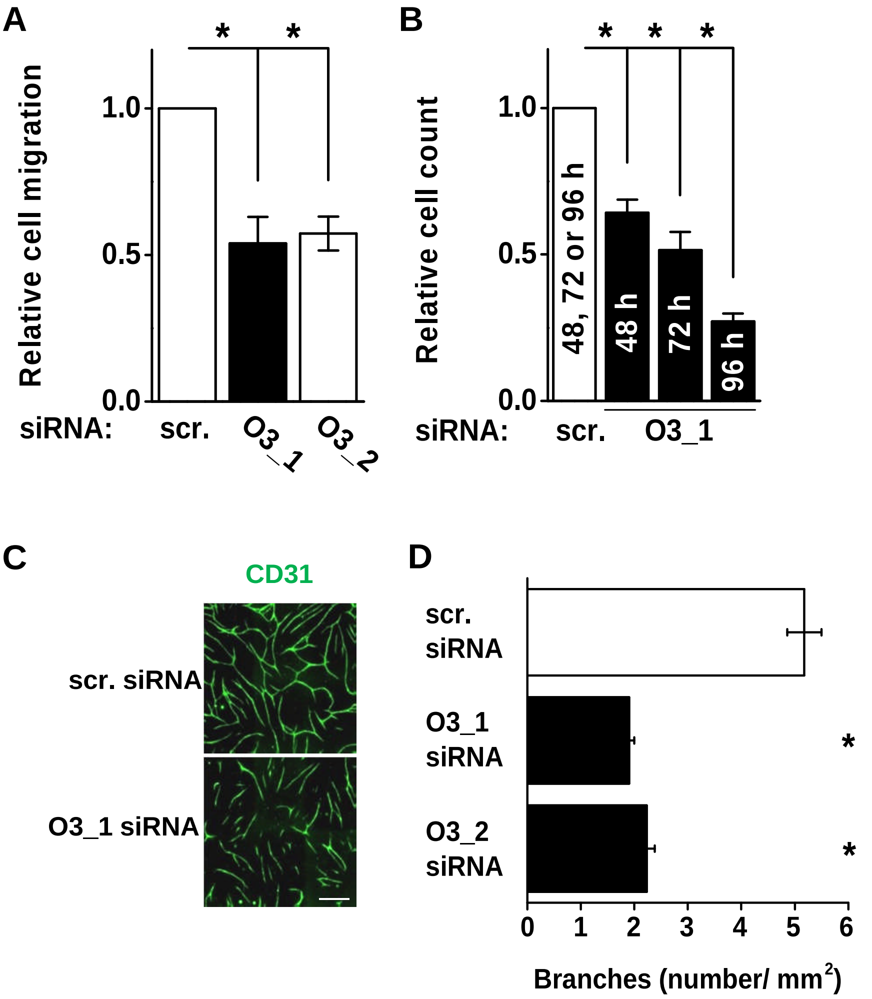

Supplemental Figure II. Additional data supporting roles of Orai3 in endothelial cell remodeling.
(A) Mean data for HUVECs in the transwell migration assay after transfection with control scrambled (scr.) siRNA or Orai3 siRNA_1 (O3_1) or Orai3 siRNA_2 (O3_2) (n=4 each).
(B) Mean data and analysis of HUVEC 48, 72 and 96 hr after transfection with Orai3 siRNA_1 and each compared with a time-matched control transfected with scr. siRNA (n=4 each).
(C) Example images for endothelial tube-formation in co-culture on fibroblasts. Images are for scr. and O3_1 siRNA transfected HUVECs labelled for CD31 (green).
(D) Mean data for the numbers of branches exemplified in **(C)** and for O3_1 and O3_2 siRNAs (n=3/N=18 each).
Data are represented as mean ± SEM; **p* < 0.05.

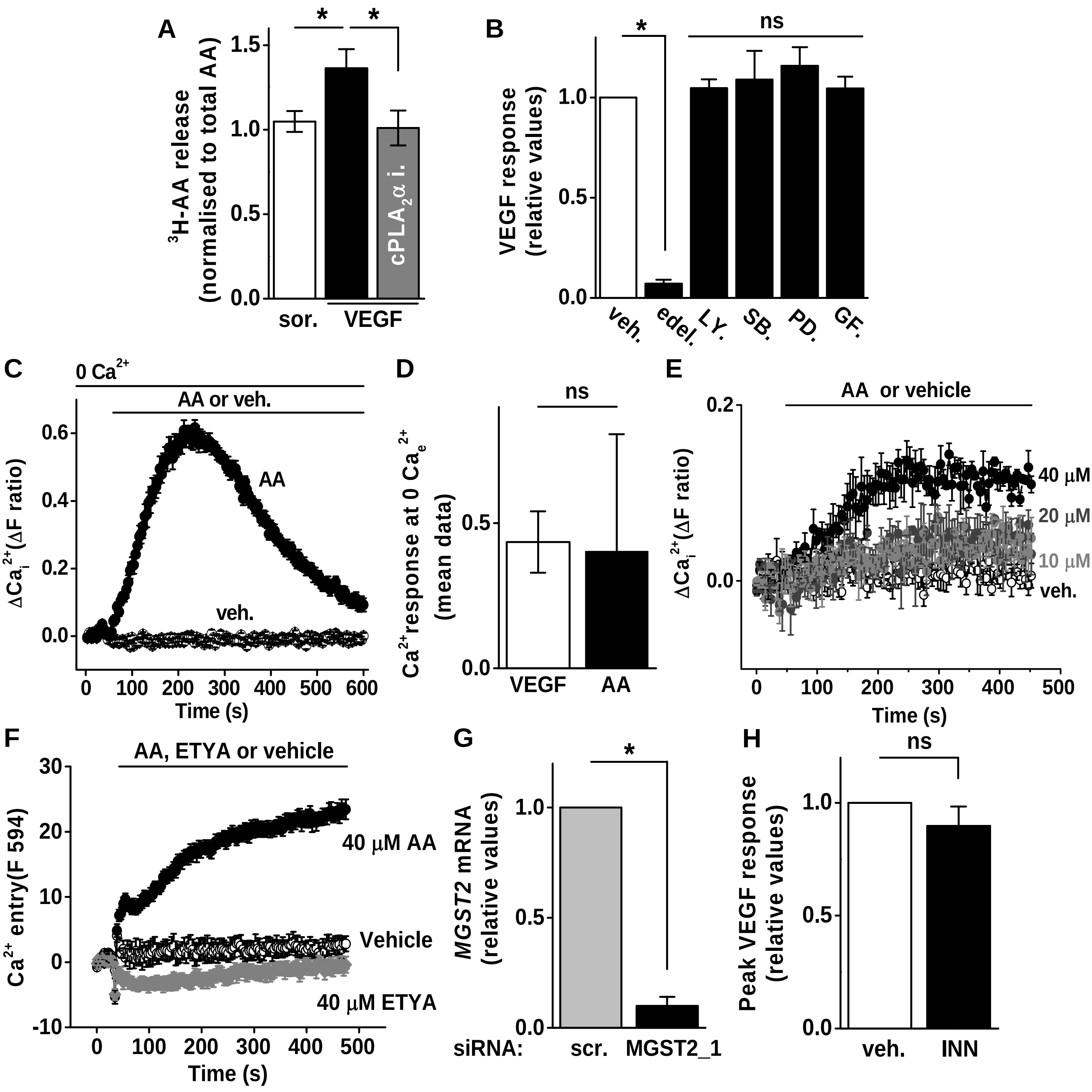

Supplemental Figure III. Additional data on the signaling pathway.

(A) Mean data for [^{3}H]-AA release from HUVECs treated with sorafenib (sor.) alone [1 μM], VEGF alone [30 ng/mL], or VEGF [30 ng/mL] plus cPLA2α inhibitor (cPLA2α i.) [1 μM] (n=6/N=16 each).

(B) Mean data and analysis of the 30 ng/mL VEGF Ca^{2+} responses from cells treated with vehicle or inhibitor. Edelfosine [10 μM] (edel., n=3/N=64), LY-294002 [10 μM] (LY., n=3/N=48), SB-203680 [100 nM] (SB., n=3/N=48), PD-98059 [100 μM] (PD., n=2/N=24), GF-109203X [1 μM] (GF., n=2/N=36).

(C) Example 40 μM AA-response from cells in the absence of extracellular Ca^{2+} (0 Ca^{2+}). The vehicle (veh.) for AA elicited no response.

(D) Mean data and analysis for the Ca^{2+} responses in the absence of extracellular Ca^{2+} from cells stimulated with VEGF [30 ng/mL] (n=7/N=112) or AA [40 μM] (n=8/N=128).

(E) Effects of 10, 20 and 40 μM AA on intracellular Ca^{2+} in HUVEC (n=3/N=72).

(F) HUVEC responses to AA [40 μM], the non-metabolizeable AA analogue ETYA [40 μM] or vehicle control (representative of n=3/N=72). Ca^{2+} entry was measured in HUVECs incubated with the non-ratiometric Ca^{2+} indicator X-Rhod-1 AM.

(G) Analysis of MGST2 mRNA abundance after transfection of HUVECs with scr. or MGST2 siRNA 1 (n=3 each).

(H) Mean data for the peak 30 ng/mL VEGF-evoked Ca^{2+} response from cells treated with vehicle or 5 μM indomethacin (INN) (n=3/N=47 each).

Data are represented as mean ± SEM; *$p < 0.05$, ns $p > 0.05$.

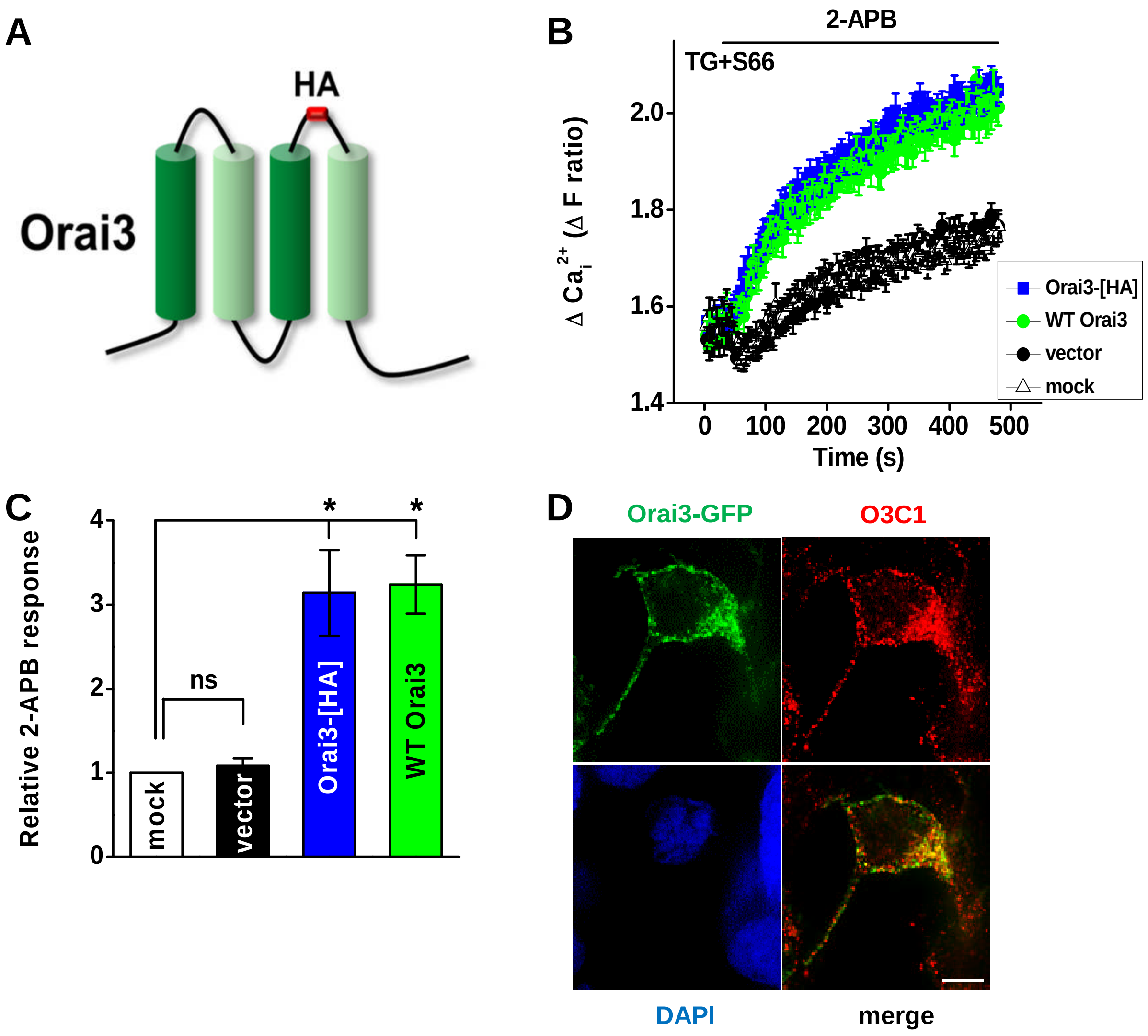

Supplemental Figure IV. Over-expression of Orai3-[HA] and Orai3-GFP in HEK 293 cells.
(A) Depiction of the Orai3-[HA] construct.
(B) Representative response to 2-APB [75 µM] from Orai3-[HA], wild-type (WT) Orai3, vector or mock transfected HEK 293 cells pretreated with 2 µM thapsigargin (TG) and in the continuous presence of 5 µM S66.
(C) Mean data for the peak 2-APB response as exemplified by the data in **(B)** (n=4/N=44 for each group).
(D) Representative images HEK 293 cells transfected with Orai3-GFP (green) and labelled with O3C1 antibody (anti-Orai3, red). Shown also is the DAPI labeling of the cell nuclei, and the merged image of Orai3-GFP and O3C1. Scale bar, 10 µm.
Data are represented as mean ± SEM; *$p < 0.05$, ns $p > 0.05$.

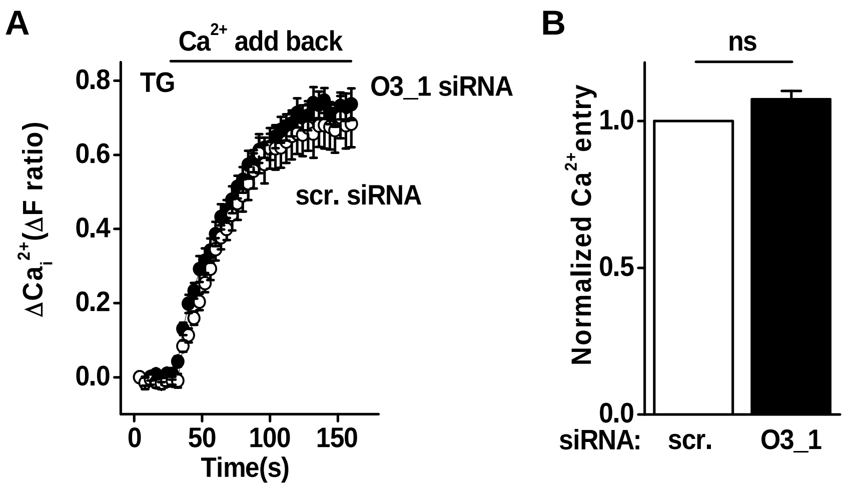

Supplemental Figure V. Orai3 does not contribute to store-operated Ca^{2+} entry in HUVECs.
(A) Representative Ca^{2+} add back response from HUVECs treated with scrambled (scr.) or Orai3 siRNA_1 (O3_1). Cells were pre-treated with 2 µM thapsigargin (TG) prior to the addition of Ca^{2+}.
(B) Mean data and analysis for the Ca^{2+} entry response exemplified in **(A)** (n=3/N=36 each) .
Data are represented as mean ± SEM; ns $p > 0.05$.

Gene	Primer 5’-3’	Predicted amplicon (bp)
Orai1	F GCACAATCTCAACTCGG<br>R GCGAAGACGATAAAGATCAG	300
Orai2	F GCATCTGGTAGACCCG<br>R ACCTCAAGTGATCCGC	232
Orai3	F CAAGGCATTGGTCTAGC<br>R AATTCAGTGTCAGAAGAGC	298
STIM1	F CTCTCTTGACTCGCCA<br>R GCTTAGCAAGGTTGATCT	276
STIM2	F TGGACCTCTAACACGC<br>R GCATACTGACGTCTACTCAA	351
TRPC1	F TTAGCGCATGTGGCAA<br>R CCACTTACTGAGGCTACTAAT	303
TRPC4	F ATTAGCTTCACGGGGT<br>R CTTCGTGGGTGACTGT	241
TRPC5	F ACATTTTAAGTTCGTTGCG<br>R ACATCGGATCCCCTTG	218
β-actin	F TCGAGCAAGAGATGGC<br>R TGAAGGTAGTTTCGTTGGATG	194
MGST2	F GAGTATTTCGGGGCACAAC<br>R GTTAGAATTGCCGCCT	303
LTC4S	F GAGTCCTGCTGCAAGCCTACTTC<br>R CGAGGAACAGCGGGAAGTAC	150

Supplementary Table I: PCR primers.
