# Supplementary material for: Orai3 Surface Accumulation and Calcium Entry Evoked by Vascular Endothelial Growth Factor
Source: Arterioscler Thromb Vasc Biol. 2015 Aug 26;35(9):1987–94. doi: 10.1161/ATVBAHA.115.305969 (PMC4548547; doi:10.1161/ATVBAHA.115.305969)
Supplement: Supplementary file 2 [file atv-35-1987-s002.pdf]

Materials and Methods

Orai3 surface accumulation and calcium entry evoked by vascular endothelial growth factor

Jing Li*[1] Alexander Bruns*[1], Bing Hou[1], Baptiste Rode[1], Peter J Webster[1], Marc A Bailey[1], Hollie L Appleby[1], Nicholas K Moss[1], Judith E Ritchie[1], Nadira Yuldasheva[1], Sarka Tumova[1], Matthew Quinney[1], Lynn McKeown[1], Hilary Taylor[1], K Raj Prasad[3], Dermot Burke[4], David O'Regan[5], Karen E Porter[1], Richard Foster[2], Mark T Kearney[1], David J Beech[†1]

[1]School of Medicine and [2]School of Chemistry, University of Leeds, Leeds, LS2 9JT, UK. [3]Department of Hepatobiliary and Transplant Surgery and [4]Department of Colorectal Surgery, St. James's University Hospital, Beckett Street, Leeds LS9 7TF, UK. [5]Yorkshire Heart Centre, Leeds General Infirmary, Great George Street, Leeds, LS1 3EX.

*Equal contribution
[†]Author for correspondence: Prof David J Beech, LIGHT Building, Clarendon Way, School of Medicine, University of Leeds, Leeds, LS2 9JT, England, UK; d.j.beech@leeds.ac.uk; Tel +44 (0) 113 34 34323.

Reagents
S66 (Synta66) (3-fluoropyridine-4-carboxylic acid (2',5'-dimethoxybiphenyl-4-yl)amide) was synthesized in Leeds. Arachidonic acid, thapsigargin and VEGF were from Sigma-Aldrich. VEGF was a homodimer of human VEGF-165 (Sigma-Aldrich catalogue number V7259). The identity and purity of AA was confirmed by mass spectrometry. Sorafenib was from Bio Vision. Merck Millipore (Calbiochem) provided the cPLA2α inhibitor (N-((2S,4R)-4-(biphenyl-2-ylmethyl-isobutyl-amino)-1-[2-(2,4-difluoro-benzoyl)-benzoyl]-pyrro-lidin-2-ylmethyl)-3-[4-(2,4-dioxothiazolidin-5-ylidenemethyl)-phenyl] acrylamide, HCl). Indomethacin was from Cayman Chemical. The protein kinase C inhibitor GF-109203X and the phospholipase C inhibitor edelfosine were from Tocris Bioscience. The p38 MAP kinase inhibitor SB-203580 and the MEK1 inhibitor PD-98059 were from Selleck Chemicals. Merck Millipore (Calbiochem) provided the phosphatidylinositol 3 (PI3) kinase inhibitor LY-294002. 2-APB was from Sigma-Aldrich. All other reagents were from Sigma-Aldrich.

Antibodies
Antibodies used for immunoblotting included anti-*β* actin (Santa Cruz, clone C4, 0.2 μg/ mL), anti-CD31 (Dako, clone JC70A, 0.2 μg/ mL), anti-CD71 (Santa Cruz, clone H68.4, 0.2 μg/ mL), anti-MGST2 (Abgent, clone RB17140, 1:1000), anti-Orai3 (custom made to a C-terminal peptide of Orai3 ([C]VAHKTDRYKQELEE), O3C1, 1:10000), anti-STIM1 (BD Biosciences, clone 44/GOK, 0.2 μg/ mL), anti-STIM1 (Santa Cruz, clone CDN3H4, 0.2 μg/ mL), anti-VE cadherin (Cell Signaling Technology, clone D87F2, 1:1000), anti-VEGFR2 (R&D Systems, 0.2 μg/ mL), anti-phospho VEGFR2 (Y1175) (Cell Signaling Technology, clone 19A10, 1:1000). Secondary antibodies included cross-adsorbed, horseradish peroxidase (HRP) conjugated donkey anti-mouse, anti-rabbit and anti-goat IgG from Jackson ImmunoResearch Laboratories. All HRP-conjugated secondary antibodies were used at 20 ng/mL. Antibodies used for immunocytochemistry included anti-CD31 (Dako, clone JC70A, 0.4 μg/mL), anti-Orai3 (O3C1, 1:500), anti-HA.11 (Covance, clone 16B12, 1 μg/mL) as well as cross-adsorbed DyLight488 and DyLight594 conjugated donkey anti-mouse and anti-rabbit IgG (all Jackson ImmunoResearch Laboratories). Fluorophore-conjugated antibodies were used at 1 μg/mL.

Cell culture

Human umbilical vein endothelial cells (HUVEC) and coronary microvascular endothelial cells (CMEC) were purchased from Lonza, cultured in EBM-2 growth medium supplemented with EGM^{TM}-2 or EGM™-2MV BulletKit™ (Lonza) respectively and passaged 2-4 times. Primary saphenous vein endothelial cells (SVEC) were isolated from segments of human saphenous vein discarded at the time of open heart surgery performed at the Leeds General Infirmary, Leeds Teaching Hospitals NHS Trust as described previously[1]. SVECs were grown in EBM-2 growth medium supplemented with EGM-2 bullet kit (Lonza) and used up to passage 3. Ethical approval was provided by the Leeds Teaching Hospitals Local Research Ethics Committee (Ref: CA01/040). Human liver endothelial cells (LEC) were isolated from patients undergoing liver resection for colorectal metastases at St James's University Hospital, Leeds Teaching Hospitals NHS Trust. Ethical approval was provided by the Leeds Teaching Hospitals Local Research Ethics Committee (Ref: 14/YH/1001). Tissue was collected immediately following resection. A section of normal liver tissue was taken from a macroscopically normal area of the surgical specimen at least 2.5cm from any tumors. The samples were stored and transported in cold EBM-2 growth medium supplemented with EGM-2 bullet kit (Lonza) on ice. Briefly, 1 g of tissue was weighed and minced in a Petri dish. The tissue was resuspended in 9 mL 0.1% collagenase and 1 mL 2.5 U/mL dispase solution and incubated for 45 min at 37°C under continuous agitation. Subsequently, the sample was passed through 100 µm and 40 µm cell strainers to remove major debris. This was followed by 2 washes in MACS buffer (PBS, 2mM EDTA, and 0.1% BSA; pH 7.2). The pellet was resuspended in 20 mL of red blood cell lysis buffer (17 mM TRIS base, 140 mM NH_4Cl) for 10 min at room temperature. Following a final wash in MACS buffer, cells were incubated with 200 µl of dead cell removal paramagnetic microbeads per $1x10^7$ cells (Miltenyi Biotec) at room temperature for 15 min. The suspension was then passed through a MACS cell separation column in a magnetic field to retain apoptotic cells, dead cells and debris. The live cell fraction was eluted and incubated with 25 µL FcR blocking reagent and 25 µL anti-CD31-conjugated paramagnetic microbeads per $1x10^7$ cells at 4°C for 15 min. This suspension was passed through a column in a magnetic field and the unbound cells eluted with buffer. Once removed from the magnetic field, the column was washed with MACS buffer to elute CD31-positive cells. The approach was validated by immunostaining. Cells were used up to passage 3. Huh7 cells were propagated in Dulbecco's Modified Eagle's Medium (DMEM)/Glutamax (Gibco) with 10 % fetal calf serum (FCS) (Sigma), 100 U/mL penicillin and 100 µg/mL streptomycin (Sigma). Normal Human Dermal Fibroblasts (NHDF) were purchased from Lonza and cultured in Fibroblast Growth Medium (FGM) (PromoCell). HEK-293 cells were propagated in Dulbecco's Modified Eagle's Medium (DMEM)/Glutamax (Gibco) with 10 % fetal calf serum (FCS) (Sigma), 100 U/mL penicillin and 100 µg/mL streptomycin (Sigma). All cells were maintained at 37 °C in a humidified atmosphere containing 5 % CO_2.

Cell migration and cell count

Migration assays were performed using modified Boyden chambers (8-µm pores; BD Biosciences). siRNA treated HUVEC were serum starved in EBM-2 for 4 hr, resuspended at 5×10^4 cells/mL in EBM-2 containing 0.4 % FCS, and loaded in the upper chamber. The lower chamber contained 0.4 % FCS supplemented with 20 ng/mL VEGF. After 4 hr the inserts were fixed with 70% ethanol for 2 min. Cells on duplicate membranes were scraped from the upper surface and migrating cells underneath were stained with hematoxylin/ eosin (H&E) and evaluated by counting in 6 randomly-chosen fields under light microscopy. For proliferation experiments, equal numbers of cells were plated at 50-60 % confluency in 6-well tissue culture plates (Corning) in EBM-2. 48 hr after transfection cells were collected by trypsinization, stained with trypan blue and the cell number determined in duplicate wells and counted at least twice with a hemocytometer.

Co-culture assays

For fibroblast-endothelial cell co-culture assays, NHDF were plated at 2×10^3 cells/well with FGM in 96-well plates (Greiner bio-one) and incubated at 37 °C in 5% CO_2. Upon confluency

of NHDF, HUVEC were seeded on top at 5.4 x 10^4 cells/well in EBM-2 supplemented with 2 % serum and 3 ng/mL VEGF. Tubules were analyzed after 7 days following fixing and staining of endothelial cells with anti-CD31 antibody. Quantification of tubule formation was conducted using IncuCyte angiogenesis v2.0 image analysis (Essen Bioscience).

In vitro **tube formation**

HUVECs in EBM-2 containing 1 % FCS and 2 ng/mL VEGF at were seeded at 2 x 10^4 cells per well in Matrigel pre-coated BD Biosciences 96-well plates. Tube formation was quantified 18-20 hr later. Digital images of endothelial tubes were obtained by bright-field light microscopy. Tube formations were measured by giving: (i) total tube length per image; and (ii) number of complete loops (circles) per image.

In vivo **Matrigel plug assay**

Ice-cold Matrigel (0.5 mL/plug; BD Biosciences) was mixed with EBM-2 containing VEGF (100 ng/mL) and 5 x 10^5 HUVECs transfected with Orai3 siRNA 1 or scrambled siRNA. Anaesthetized 6 to 8-week-old mice were injected subcutaneously into the flanks. After 7 days Matrigel plugs were dissected from anaesthetized mice, washed with phosphate-buffered saline (PBS), fixed in 10 % paraformaldehyde, embedded in paraffin, sectioned, and stained with (H&E). Digital images were obtained by bright-field light microscopy. Male CD1 nude mice (Charles River) were used in accordance with ethical approval under UK Home Office license.

Shear stress

For shear-stress experiments HUVECs were treated as described previously[1]. Briefly, 2 x 10^5 HUVECs were plated on a 6-well tissue culture plate and cultured for 24 hr prior to being placed on an orbital shaker (210 rpm) for 16 hr. The swirling motion of medium around the edges of the wells produced tangential shear stress, which results in cell elongation and alignment along the edges[1]. As a control, HUVECs were cultured in parallel under static conditions.

Short interfering (si) RNAs and molecular biology

HUVECs, CMECs, SVECs and LECs were transfected at 90 % confluence with 20 nM siRNA using Lipofectamine 2000 in OptiMEM (Gibco) as per the manufacturer's instructions (Invitrogen). Medium was replaced after 3-4 hr and cells were used for experimentation 48 hr after transfection. Ambion provided all siRNAs. The sequences for Orai3 were CCGCUACAAGCAGGAACUAfor siRNA_1 and GCUACAAGCAGGAACUAGA for siRNA_2. Unless otherwise stated Orai3 siRNA_1 was used. siRNA sequences for MGST2 were CGGCCUGUCAGCAAAGUUA for siRNA_1 and GUUUUAUCCUAUAUUCAUA for siRNA_2. Unless otherwise stated MGST2 siRNA_1 was used. Scrambled siRNA 1 was used as a control. Full-length cDNA for Orai3 (accession number BC015555) was purchased from Geneservice (Source Bioscience LifeSciences) and sub cloned into pcDNA6/V5-His (Invitrogen) at *Eco*RI/*Xho*I sites. For the generation of Orai3-[HA] an HA-tag (TATCCATATGATGTTCCAGATTATGCT) was inserted into the 2nd extracellular loop of Orai3 between S214 and A217. Orai3 in pcDNA6 was linearized by inverse PCR using 5' GCAGCACCGTCCCAGG and AGAGGACCGTGGGAGATTGGAAGCTG primer. The tag was amplified using primers that overlap with the inverse PCR: 5' AATCTCCCACGGTCCTCTTATCCATATGAT and 5' CCTGGGACGGTGCTGCAGCATAATCTGGAAC. The PCR fragments were gel-purified and recombined using the Clontech In-fusion kit as per the manufacturers' instructions (Takara-Bio). mCherry-Orai1 was a gift from S. Muallem. Full length cDNA of Orai3 was cloned in pEGFP-N1 vector at BamHI/XbaI sites.

Quantitative real-time RT-PCR

Total RNA was extracted using Tri-reagent followed by DNase I (Ambion) treatment. 1 µg of total RNA was used for reverse transcription (RT) using oligo-dT primers (Ambion) and AMV RT enzyme (Ambion). Real-time PCR was conducted using a Roche LightCycler. Specificity of PCR was verified using reactions without RT enzyme (-RT) and by melt-curve analysis. PCR primers are given in Online Table I. PCR products were analyzed on 2 % agarose gels and sequenced to confirm identity.

Intracellular Ca^{2+} measurement

Cells were incubated with fura-2AM [2 µM] (Thermo Fisher Scientific), or X-Rhod-1 AM [2 µM] (Thermo Fisher Scientific) for 60 min at 37 °C followed by a 30 min wash at room temperature (RT: 21± 2 °C). Where indicated, S66 and TG were added at room temperature 15 min before taking Ca^{2+} measurements. All other inhibitors were added during the 30 min wash. All inhibitors were present during Ca^{2+}-measurements. Measurements were made at room temperature on a 96-well plate reader (FlexStation, Molecular Devices). The change (Δ) in intracellular Ca^{2+} concentration was indicated as the ratio of fura-2 emission (510 nm) intensities for 340 nm and 380 nm excitation (F ratio). Wells within columns of the 96-well plate were loaded alternately for test and control conditions. The recording solution (standard bath solution, SBS) contained (mM): 130 NaCl, 5 KCl, 8 D-glucose, 10 HEPES, 1.2 $MgCl_2$, 1.5 $CaCl_2$, titrated to pH 7.4 with NaOH. When Ca^{2+}-free extracellular solution was used, $CaCl_2$ was omitted; for Ca^{2+} add-back, Ca^{2+} was 0.3 mM.

Arachidonic acid release

HUVEC were grown on 0.1 % gelatin-coated 6-well plates to the required density followed by addition of 0.5 µCi [^{3}H]-AA (PerkinElmer) per well. After 24 hr, cells were washed 3 times with SBS followed by a 30 min recovery period in SBS. Cells were subsequently treated with sorafenib, cPLA2α inhibitor, or vehicle control (DMSO) for 30 min, washed with SBS, and incubated for 20 min in SBS containing 0.3 % fatty acid-free bovine serum albumin with or without VEGF and with or without cPLA2α inhibitor. The amount of released [^{3}H]-AA in cleared medium and the total radioactivity from cells solubilized in 0.1 % Triton X-100 were quantified by liquid scintillation counting.

Immunocytochemistry, image collection and analysis

For immunocytochemical staining of endogenous proteins, confluent HUVECs were washed twice in SBS, serum-starved for 20 min, and pre-incubated with sorafenib, vehicle control (DMSO) or, where indicated, with cPLA2α inhibitor for 10 min. Cells were then treated with VEGF or AA for 5 and 10 min, respectively, in the presence or absence of sorafenib or cPLA2α inhibitor where indicated. Cells were fixed with pre-warmed 2 % paraformaldehyde for 5 min, permeabilised with 0.1 % TritonX-100 for 5 min at RT, and non-specific sites were blocked using 5 % donkey serum/PBS for 60 min at room temperature. Primary antibody incubation was in 1 % donkey serum/PBS overnight at 4°C. Secondary antibody incubation was in 1 % donkey serum/PBS for 60 min at room temperature. Cells were counter-stained with DAPI and mounted with Prolong Gold Antifade Reagent (Invitrogen). Cells were visualized using a LSM 700 microscope (Zeiss) using a 63x/1.4 Oil DIC Plan Apochrom, M27at objective. For labeling of Orai3-[HA], HUVECs were grown on glass coverslips to confluency and transfected with 250 ng DNA per well using Lipofectamine 2000. Cells were studied 6 hr after transfection in order to minimize the risk of over-expression. For experiments with mCherry-Orai1, 24 hr after transfection cells were essentially treated as cells transfected with Orai3-[HA]. For immunocytochemistry experiments with HEK293 cells, cells were transfected at 70% confluency with 500 ng Orai3-GFP per well using Lipofectamine 2000. 24 hr after transfection cells were, as described above, fixed with pre-warmed 2 % paraformaldehyde for 5 min, permeabilised with 0.1 % TritonX-100 for 5 min at RT, and non-specific sites were blocked using 5 % donkey serum/PBS for 60 min at room temperature. Primary antibody incubation was in 1 % donkey serum/PBS overnight at 4°C. Secondary antibody incubation was in 1 % donkey serum/PBS for 60 min at room temperature. Cells were counter-stained with DAPI and mounted with Prolong Gold Antifade

Reagent (Invitrogen). Cells were visualized using a DeltaVision deconvolution system (Applied Precision Instruments, Seattle, WA) with Olympus IX-70 inverted microscope using a 40x UPLAN objective (NA 1.35). Images were collected at room temperature using a Roper CoolSNAP HQ CCD camera with SoftWoRx acquisition and analysis software. For analysis, the integrated density of fluorescence along the cell outline in a 5-pixel wide corridor as a fraction of the total cell perimeter was measured using the raw images.

Cell surface biotinylation

HUVECs were grown in 10 cm dishes to confluency, washed twice in SBS, serum-starved for 20 min, and pre-incubated with sorafenib or vehicle control (DMSO) for 10 min. Cells were then treated in SBS with VEGF or AA for 5 and 10 min, respectively, in the presence or absence of sorafenib. After the respective treatment, cells were chilled on ice, washed twice with ice-cold PBS containing 1 mM Ca^{2+} and 1 mM magnesium (Mg^{2+}) and biotinylated for 30 min using 0.15 mg/mL EZ-Link Sulfo-NHS-Biotin (Pierce) in PBS/ 1 mM Ca^{2+}/ 1 mM Mg^{2+} at 4°C with gentle rocking. Subsequently, cells were washed 3 times for 5 min with ice-cold TBS (137 mM Sodium Chloride, 100 mM Tris; pH 7.4)/ 1 mM Ca^{2+}/ 1 mM Mg^{2+} to quench excess biotin, and twice with ice-cold PBS/ 1 mM Ca^{2+}/ 1 mM Mg^{2+}. Cells were lysed in Standard Lysis Buffer (SLB) containing 10 mM Tris, pH 7.4, 150 mM NaCl, 0.5 mM EDTA, 0.5 % NP-40, 5 % glycerol, Protease and Phosphatase Inhibitor Cocktail (Sigma-Aldrich). Biotinylated proteins were concentrated using streptavidin-agarose (Pierce) overnight at 4 °C. Proteins were resolved as described below. Input was 15 µg. Pull down representing the cell surface fraction was 1 mg.

Immunoblotting

HUVECs were harvested in SLB. Proteins were quantified using the RC DC protein assay (BioRad). Equal protein amounts were loaded on 10 % SDS gels and resolved by electrophoresis. Samples were transferred to a PVDF membrane and labeled overnight with the indicated antibodies. HRP-conjugated secondary antibodies and SuperSignal West Pico, West Femto (Thermo Fisher) or West Star ηC (Cyanagen) detection reagents were used for visualization.

Data analysis

All images and densities of protein bands were analyzed using ImageJ software (NIH). Statistical analysis was performed using OriginPro (OriginLab Corporation). Averaged data are presented as mean $\pm$SEM. Where data were produced in multiple pairs (test and control) and normalized, bar charts show a single control bar even though each test data set had its own matched control. Prior to statistical analysis data was tested for normality and equality of variance. Paired data were compared statistically using *t* tests. Data sets with more than 2 groups were compared by ANOVA and a post-hoc Bonferroni test. Statistical significance was indicated by *(p<0.05) and no significant difference by ns (p>0.05). The number of independent experiments is indicated by n. For multi-well assays, the number of replicate wells is indicated by N.

References

1. Li J, Hou B, Tumova S, Muraki K, Bruns A, Ludlow MJ, Sedo A, Hyman AJ, McKeown L, Young RS, Yuldasheva NY, Majeed Y, Wilson LA, Rode B, Bailey MA, Kim HR, Fu Z, Carter DA, Bilton J, Imrie H, Ajuh P, Dear TN, Cubbon RM, Kearney MT, Prasad KR, Evans PC, Ainscough JF, Beech DJ. Piezo1 integration of vascular architecture with physiological force. *Nature*. 2014;515:279-282
